# Sustained improvement in dark-adapted sensitivity but not BCVA after voretigene neparvovec treatment in a mainland Chinese child with biallelic *RPE65*-associated LCA2: a case report

**DOI:** 10.3389/fphar.2026.1888954

**Published:** 2026-09-03

**Authors:** Yue Ren, Shu Liu, Yanbo Liu, Di Wang, Jia Rong, Wensheng Li, Chunhui Zheng, Ling Xu, Jun Li, Zhuoshi Wang, Jijing Pang

**Affiliations:** 1 Shenyang He Eye Specialist Hospital, Shenyang, China; 2 He University, Shenyang, China; 3 Xiamen Eye Center and Eye Institute of Xiamen University, School of Medicine, Xiamen, China; 4 Shanghai Aier Eye Hospital, Aier Eye Hospital Group Co. Ltd., Shanghai, China

**Keywords:** case report, gene therapy, leber congenital amaurosis, luxturna, RPE65

## Abstract

Inherited retinal dystrophies (IRDs) represent heterogeneous genetic eye disorders frequently linked to *RPE65* mutations. Voretigene neparvovec (VN, Luxturna), an AAV2-mediated gene therapy, remains the sole approved drug for biallelic *RPE65*-associated IRDs. This study presents 3-, 6- and 22-month follow-up findings of the first mainland Chinese patient with compound heterozygous *RPE65*-related Leber congenital amaurosis type 2 (LCA2) receiving sequential bilateral subretinal Luxturna injections. Binocular full-field stimulus threshold (FST) improvement was documented, and rod-dominant visual function improvement was verified via chromatic threshold difference analysis. No treatment-related adverse events were noted at the 3-month follow-up, while bilateral chorioretinal atrophic lesions, classified as treatment-emergent ocular findings with undetermined causal correlation to gene therapy, were detected at the 6-month visit, and these lesions further enlarged at the 22-month follow-up. Long-term assessment confirmed persistent improvement in dark-adapted visual function but not best-corrected visual acuity (BCVA). This single case demonstrates the favorable sustained dark-adapted rod function improvement of voretigene neparvovec in this Han Chinese patient with LCA2. Although obvious improvement in nyctalopia after early gene therapy has been observed in this patient, all FST data in this study are binocular integrated measurements that cannot be separated to evaluate individual monocular function. Further controlled cohort studies enrolling more Han Chinese participants are still required to verify whether early intervention provides more prominent and definitive relief of nyctalopia for patients carrying biallelic *RPE65* variants.

## 1 Introduction

Inherited retinal dystrophies (IRDs) are a group of rare blinding diseases that commonly affect children and young adults (Georgiou et al., 2024). Mutations in over 338 different genes are associated with IRDs, including biallelic *RPE65* mutation-related retinal dystrophy (Ziccardi et al., 2019; RetNet, 2026; Travis et al., 2007; Kang and Scott, 2020). Different mutations in *RPE65* gene can result in diverse phenotypes. The main clinical symptoms include night blindness and nystagmus, with considerable variability in daytime vision with/without progressive decline. Patients also experience gradual constriction of VF and continuous photoreceptor loss, eventually leading to blindness (Thompson et al., 2000; Kumaran et al., 2017). Based on the severity and characteristics of clinical manifestations, individuals with biallelic *RPE65* mutations may receive different clinical diagnoses. The most severe type is usually called LCA2 with complete night blindness, little daytime vision and nystagmus with or without oculodigital sign months to years from birth; the moderate type is often termed as severe early-onset childhood retinal dystrophy (EOCRD) or early-onset severe retinal dystrophy (EOSRD) with complete night blindness, moderately reduced daytime vision with or without nystagmus months from birth; mild cases are referred to as early-onset retinitis pigmentosa or retinitis pigmentosa type 20 with complete night blindness, relatively good daytime vision from birth but deteriorating from school age; in rare instances, it can present as fundus albipunctatus or congenital stationary night blindness with complete night blindness and almost normal daytime or central vision throughout the life (Wolfram et al., 2025). All these types exhibit typical night blindness with autosomal recessive inheritance (Thompson et al., 2000). In very rare cases, a dominant mutation in a single *RPE65* allele can also cause retinitis pigmentosa type 87 (Bowne et al., 2011) with choroidal involvement, which is not an indication for VN and is not discussed here, as genetic testing can distinguish between single and biallelic *RPE65* mutations. Due to the diversity of genetic backgrounds and disease manifestations, early clinical diagnosis of some IRDs caused by biallelic *RPE65* mutations can be challenging, and effective treatments have been lacking. But the gene diagnosis with pathogenic or likely pathogenic biallelic *RPE65* mutations from both parents is the unique indication of Luxturna application. Luxturna is a recombinant adeno-associated virus serotype 2 (AAV2) vector-based gene therapy designed to restore RPE65 enzyme and ultimately rhodopsin, thereby improving visual function in both children and adults. It delivers a functional copy of the human *RPE65* gene into viable RPE cells via a single subretinal injection (Russell et al., 2017; LUXTURNA, 2026; Apte, 2018). Before VN was approved, no treatments were available to restore the vision, slow or halt the progression of vision loss in these diseases (Thompson et al., 2015). VN has been approved in several countries with high *RPE65* mutation detection rates, including the USA (LUXTURNA, 2017) and the EU (Novartis, 2018), for treating hereditary retinal dystrophy caused by biallelic *RPE65* mutations in patients with vision loss but sufficient viable retinal especially photoreceptor cells (Keeler and Flotte, 2019). However, the high cost of VN and limited understanding of gene therapy among some Chinese individuals have precluded its clinical application in China. Consequently, the safety and efficacy of VN in mainland Chinese patients remained unclear before the present case, and VN was not permitted for use in mainland China prior to this patient’s treatment. In a cohort study including 1,434 Chinese IRD patients using targeted next-generation sequencing (NGS) panel of 586 genes, 74.55% (n = 1,069) received a genetic diagnosis; among them, only 20 patients (17 families) carried biallelic RPE65 mutations, which accounted for 1.87% of all genetically diagnosed IRD patients, ranking as the 14th most common causative gene; 10 novel variants were identified including 4 missense, 4 splicing and 2 splicing variants (Gao et al., 2021). The low overall detection rate of *RPE65* mutations among Chinese LCA probands (1%–11% across independent cohorts) creates unique clinical screening and eligibility challenges for Luxturna gene therapy within mainland China, reinforcing the translational value of this first domestic treated case.

Here, we report the clinical outcomes of the first patient from mainland China treated with VN. Our results demonstrate the safety and efficacy of VN in treating LCA2 caused by biallelic *RPE65* mutations in the Chinese Han population. This finding brings new hope to Chinese patients with IRDs due to biallelic *RPE65* mutations and advances the development of genomic precision medicine worldwide.

## 2 Case presentation

### 2.1 Patient information

The patient is a Han Chinese male born in May 2014. His parents observed that his eyes could not fixate or follow objects after birth. By the age of two, he was found to exhibit nystagmus, night blindness, and a positive oculodigital sign (eye-pressing sign).

**FIGURE 1:**
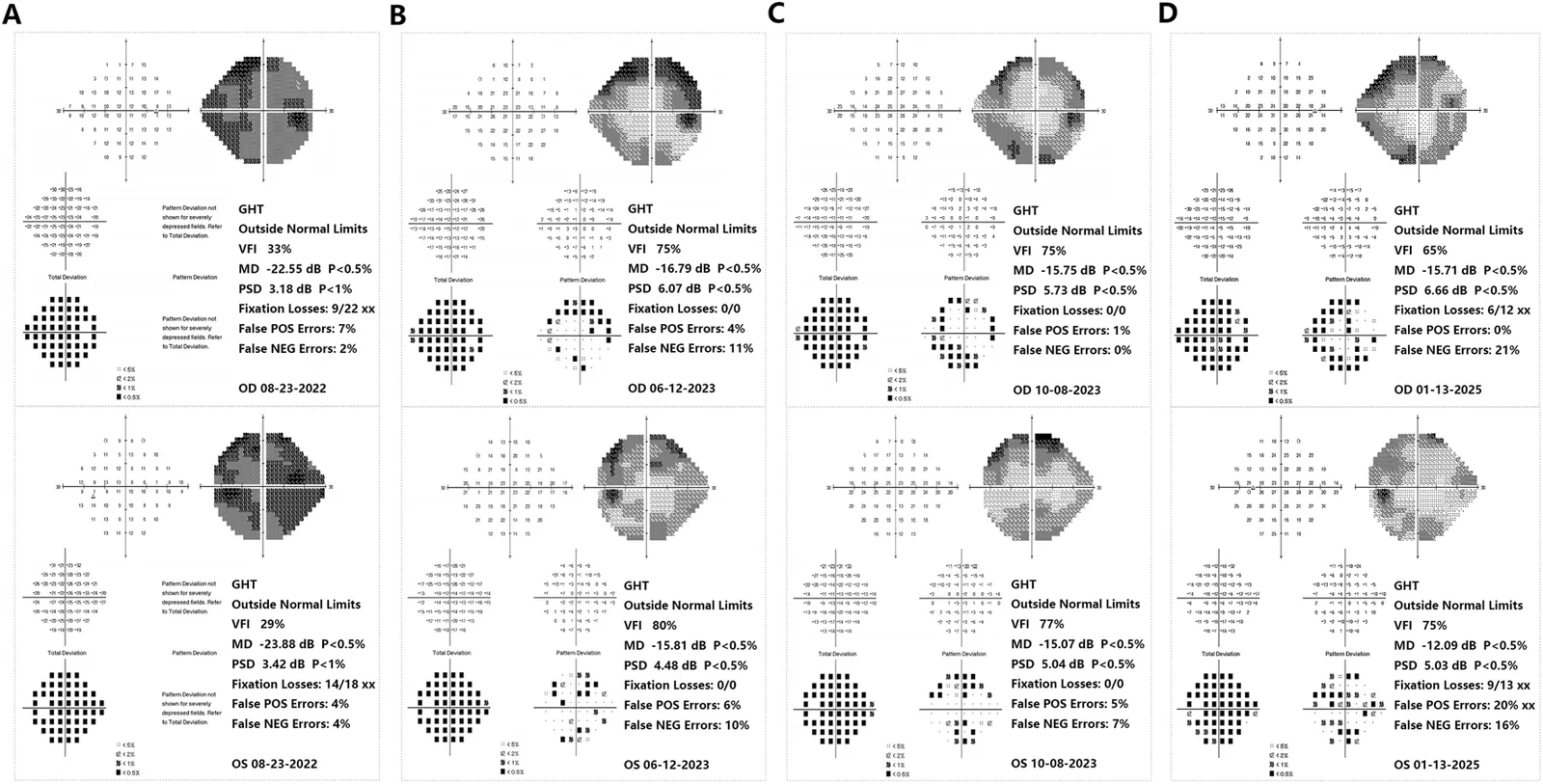
Visual field outcomes of visual filed following subretinal Luxturna gene therapy. **(A)** Pre-treatment baseline. MD was severely impaired bilaterally; OD: −22.55 dB, OS: −23.88 dB. **(B)** 3-month after bilateral subretinal Luxturna injections. OD: rose markedly to −16.70 dB; OS: increased to −15.81 dB. **(C)** 6-month after bilateral subretinal Luxturna injections. MD remained stably maintained; OD was −15.75 dB; OS was −15.07 dB. **(D)** 22-month after bilateral subretinal Luxturna injections. OD: decreased to −15.71 dB; OS: moderately declined to −12.09 dB. VF: visual field; MD: mean deviation; OD: right eye; OS: left eye.

The patient underwent genetic testing in October 2017 and August 2022; both results indicated one likely pathogenic mutation inherited from his father and one pathogenic mutation from his mother in the *RPE65* gene. Based on these findings, he was diagnosed with LCA2. His BCVA was 0.8 logMAR in the right eye and 1.0 logMAR in the left eye. Baseline clinical testing revealed constricted visual field (VF), with a mean deviation (MD) of −22.55 dB in the right eye and −23.88 dB in the left eye (Figure 1A). The patient also showed severely reduced light sensitivity (Table 1). Anterior segment examination with a slit lamp revealed almost no abnormalities. The corneas were transparent, with normal anterior chamber depth and clear iris texture. The pupils were round, and the pupillary light reflex was slightly delayed. The lenses were clear. Intraocular pressure was 16 mmHg in both eyes (Table 2). The patient was treatment-naive for ophthalmic diseases.

**TABLE 1:** Binocular FST results at pre-treatment, 3-, 6- and 22-month post-treatment.

Test time point	Light sensitivity (log cd·s/m^2^)		
	White	Blue	Red
Pre-treatment baseline	−1.905 (0.95 dB)	−2.361 (−3.61 dB)	−1.319 (6.81 dB)
Post-treatment			
3-month	−5.013 (−30.13 dB)	−5.770 (−37.7 dB)	−3.173 (−11.73 dB)
6-month (8-h dark adaptation)	−6.530 (−45.3 dB)	−7.116 (−51.16 dB)	−4.514 (−25.14 dB)
22-month (2-h dark adaptation)	−6.344 (−43.44 dB)	−6.976 (−49.76 dB)	−4.424 (−24.24 dB)
Age-matched normal control (2-h dark adaptation)	−7∼-6 (−50∼-40 dB)	−9∼-7 (−70∼-50 dB)	−5∼-4 (−30∼-20 dB)

All FST values represent binocular test results from both eyes.

**TABLE 2:** Summary of ocular signs at each follow-up time point.

Ocular parameters	Baseline	3-month	6-month	22-month
Intraocular pressure (IOP, mmHg): OD/OS	16/16	13/14	15/13	15/18
Eye inflammations	-	-	-	-
Best corrected visual acuity (logMAR): OD/OS	0.8/1.0	0.6/0.7	0.6/0.7	0.8/0.9
Cataract	-	-	-	-
Retinal tear, retinal detachment, hemorrhage in the retina	-	-	-	-
Macular hole	-	-	-	-
Dellen (thinning of corneal stroma)	-	-	-	-
Chorioretinal atrophy	-	-	Depigmentation(+)	Depigmentation(++)

**FIGURE 2:**
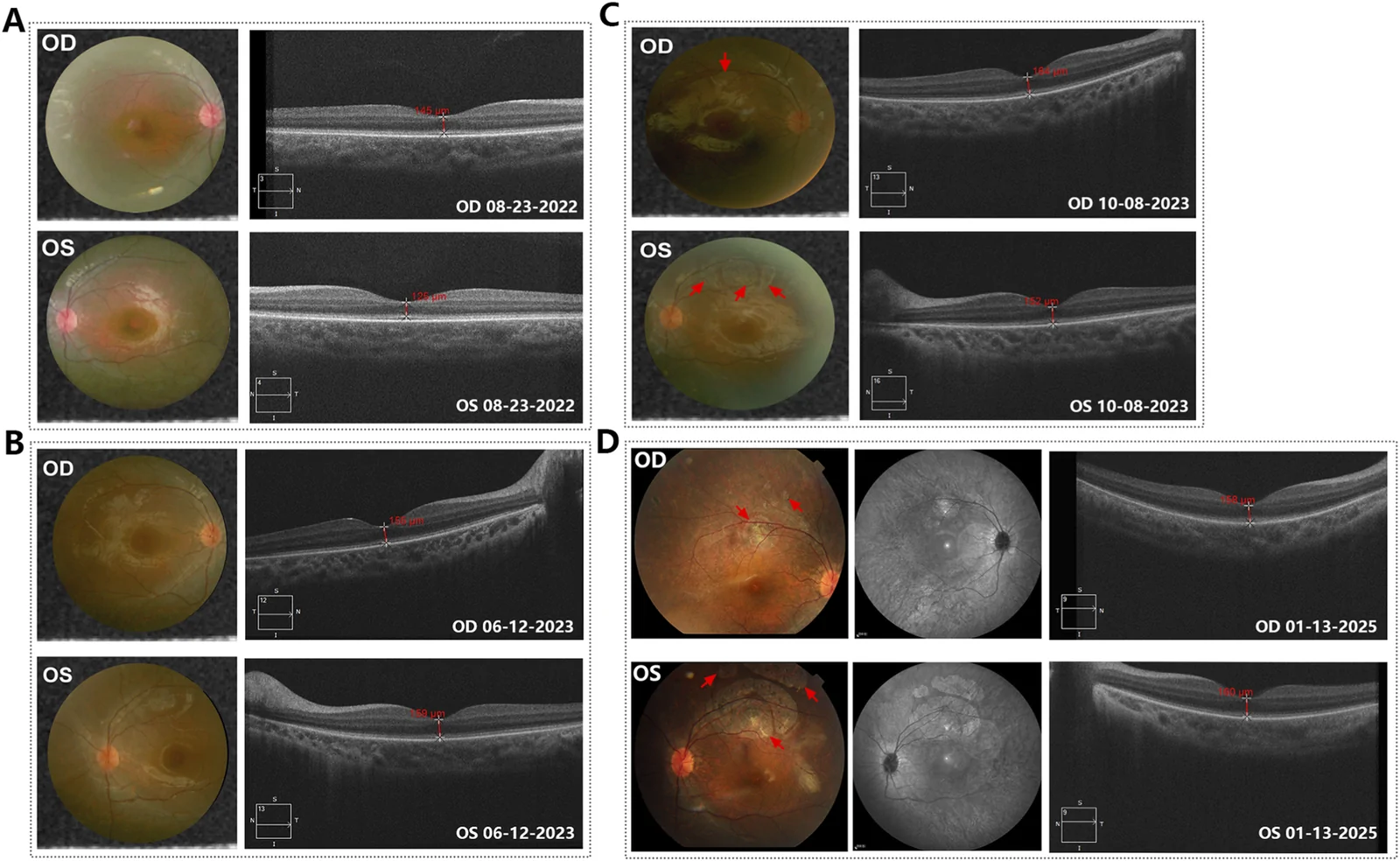
Multimodal imaging before and after treatment. **(A)** Pre-treatment baseline of fundus photography and OCT examination. Focal pigmentary disturbance and mottling in both eyes. The foveal thickness (FT) was 145 μm in OD and 125 μm in OS. **(B)** Fundus photography and OCT images at 3-month post-treatment. The FT was 155 μm in OD and 159 μm in OS. **(C)** Fundus photography and OCT images at 6-month post-treatment. The FT was 164 μm in OD and 152 μm in OS. Fundus imaging revealed depigmented chorioretinal atrophic lesions at the bilateral injection sites (red arrows). OD showed a 0.5-papillary diameter (PD) round atrophic lesion adjacent to the superotemporal vascular arcade. OS presented a 2 PD round atrophic lesion at the central injection site, surrounded by two peripheral depigmented patchy lesions of 1 PD each. **(D)** Multimodal imaging with infrared reflectance, fundus photography and OCT examinations at 22-month post-treatment. The FT was 158 μm in OD and 160 μm in OS. Multimodal imaging with infrared reflectance and fundus photography demonstrated progressive coalescence and expansion of bilateral chorioretinal atrophy. OD exhibited a 1 PD nummular atrophic lesion (red arrow) located approximately 2 PD superotemporal to the fovea along the vascular arcade, accompanied by punctate depigmented lesions. OS showed a horizontally oval 6 PD depigmented lesion approximately 1.5 PD from the fovea at the superotemporal arcade, with two superior patchy lesions measuring 1/3 PD and 2 PD, respectively. Red arrows indicate chorioretinal atrophic lesions. With infrared imaging, chorioretinal atrophic lesions were visible within the bilateral injection sites, spatially consistent with the lesions identified on fundus photography.

Dilated fundus examination showed normal optic discs with clear margins and a flat retina. In the macular region, there was slight darkening and pigmentary disturbances. Slightly narrowed retinal blood vessels were observed throughout the retina, along with scattered salt-and-pepper-like small white mottling and black pigment granules deposited in the peripheral retina (Figure 2A). Optical coherence tomography (OCT) revealed blurring of retinal layers and loss of the ellipsoid zone in both eyes. Foveal thickness (FT) was reduced: 145 μm in the right eye and 125 μm in the left eye before treatment (Figure 1A).

### 2.2 Indication to Luxturna

While the FDA-approved prescribing information only states the general principle that “viable retinal cells must be present as determined by the treating physician”. In actual clinical access, to ensure that patients still have sufficient target cells (primarily RPE and photoreceptor cells) to produce a therapeutic effect, the patients should meet one of the following three objective criteria: 1.) OCT: Regions of posterior pole have retinal thickness >100 μm; or 2.) Fundus Examination: Regions of the posterior pole with no atrophy or pigmentary degeneration ≥3 papillary disc areas; or 3.) VF Testing: Residual visual field remains within 30° of fixation.

When we applied the Luxturna through an official Novartis Managed Access network, we provided the baseline data of this patient, which meet all the above 3 objective criteria as we also showed in Figures 1, 2.

### 2.3 Gene sequencing results

Genetic testing was performed via an ophthalmic targeted next-generation sequencing gene panel, proband genetic sequencing was outsourced to a certified third-party clinical genetic testing laboratory using an ophthalmic targeted gene panel assay. Two mutations in the *RPE65* gene (reference transcript ID: NM_000329.3): c.1409C>T (p.Pro470Leu) and c.637C>T (p.Gln213Ter). The c.1409C>T mutation was determined to be a known pathogenic mutation in accordance with the ACMG guidelines, supported by criteria PM2_Supporting, PM3_Strong and PP3_Strong; paternal inheritance of this mutation was verified by Sanger sequencing. The c.637C>T mutation is a typical nonsense mutation that introduces a premature stop codon at amino acid position 213 (Gln213Ter), resulting in premature termination of the peptide chain. This mutation was classified as pathogenic per the ACMG guidelines, supported by criteria PVS1, PM2_Supporting and PM3_Strong, and maternal inheritance was confirmed via Sanger sequencing. Both parents have no related symptoms or signs at all.

Both mutations disrupt protein function, which are non-polymorphic, and exhibit extremely low allele frequencies in the general population (reference databases: 1000 Genomes and dbSNP). The segregation pattern of the two mutations within the family, together with perfect concordance with the clinical phenotype, strongly confirms a diagnosis of autosomal recessive LCA2 caused by biallelic pathogenic mutations in *RPE65*.

### 2.4 Outcome measures

To evaluate the efficacy of VN by comparing the changes in efficacy indicators after treatment with the baseline before treatment, including BCVA, FST, mean deviation (MD), OCT, slit-lamp microscopy, fundus photography. All tests were performed monocular except FST because the patient could only get stable high quality baseline data by binocular test.

#### 2.4.1 BCVA

Visual acuity was measured and expressed originally in decimal notation using the Chinese standard logarithmic E-type visual acuity chart and the standard testing distance for BCVA is 5 m. After standardized refractive correction, letter scores were converted to log of the minimum angle of resolution (logMAR).

#### 2.4.2 FST

FST was performed using a Diagnosys Epsilon system with the ColorDome stimulator (Diagnosys LLC, Lowell, MA, USA) to test the potential physiological function of photoreceptors primarily affected by biallelic *RPE65* mutations. Rod photoreceptor sensitivity was measured through the use of white and blue stimuli, and cone photoreceptor sensitivity was measured through the use of red stimuli (Gao et al., 2021; Shi et al., 2024; Jolly et al., 2024). FST is unaffected by nystagmus and suitable for evaluating a wide spectrum of visual deficits. At 3-month post-treatment, FST was dark-adapted 2-h according to the standard protocol; at 6-month post-treatment, considering the insufficient restoration of visual function/rhodopsin, the dark adaptation was extended to 8-h. In the FST testing, luminance was measured on a decibel (dB) scale, where baseline of 0 dB corresponded to a luminance of 0.01 cd·s/m^2^. White, blue, and red stimuli were used for FST testing, thresholds were measured in triplicates for each color and the averaged result from the three tests were used for each color in order to determine receptor type mediating threshold. Combining our in-house normative FST data and published literature, we established the normal reference range for binocular FST (Shi et al., 2024; Jolly et al., 2024) showed in Table 1.

The 8-h dark-adaptation FST test at 6-month post-treatment was only an exploratory assessment and should not be longitudinally compared with standard 2-h dark-adaptation data collected at the 3-month and 22-month post-treatment visits.

The luminance threshold output of the FST system was converted to decibel (dB) units following the standard conversion formula built into the Diagnosys Epsilon ColorDome device, with the factory-set reference luminance defined as 0 dB = 0.01 cd・s/m^2^:

$$dB=10\times \log_{10}\left(\frac{\text{threshold}\;\text{luminance}}{0.01}\right).$$

An important limitation of the present study is that we failed to obtain reliable monocular FST data by occluding the fellow eye at baseline and follow-up visits. All FST outcomes merely reflect combined binocular sensitivity dominated by the better-seeing eye, and cannot quantify independent rod function of the right or left eye separately. For this reason, we did not conduct correlational analysis between binocular FST improvements and monocular BCVA or MD changes to avoid misleading interpretation.

#### 2.4.3 Humphrey visual fields

The patient underwent a visual field test on Humphrey’s Field Analyzer 750i (Carl Zeiss-Humphrey Systems, Dublin, CA, USA) using the central 24-2 SITA-Fast strategy. The visual field test was considered satisfactory if false-positive and false-negative errors did not exceed 15% and fixation errors did not exceed 20%. Mean Deviation (MD) is a global index in visual field testing of Humphrey Field Analyzer that quantifies the overall departure of the patient’s measured light sensitivity from the age-corrected normal values. MD = 0 would mean the patient’s overall sensitivity matches the age-corrected normal perfectly. Negative values indicate reduced sensitivity (worse than normal).

#### 2.4.4 OCT

Optical coherence tomography (Zeiss Cirrus HD-OCT 5000) of the macula was performed to determine the presence of sufficient viable photoreceptor cells to qualify for gene therapy.”

### 2.5 Efficacy assessment

In March 2023, the patient underwent bilateral sequential subretinal VN administrations, with the right eye treated first and the left eye 1 week later, performed by the same surgeon and operating team at AI Watany Eye Hospital. Immunomodulatory drugs were given before and after injections to minimize potential immune responses to the viral vector and transgene. Each eye received a single subretinal injection of VN. The standardized protocol from Novartis was applied as follows: the total vector genome dose per eye was 1.5 × 10^11^ vg, delivered in an injection volume of 0.3 mL. The original stock concentration was 5 × 10^12^ vg/mL and diluted 1:10 before administration to reach the working concentration. Surgery consisted of three-port pars plana vitrectomy under general anesthesia. Subretinal injection guided by intraoperative OCT, with injection sites placed along the superior vascular arcades at 2 mm away from the fovea to avoid direct detachment to the fovea. The intraoperative OCT showed that the injection blebs did not reach the fovea during the procedures. No severe intraoperative complications including massive hemorrhage or large retinal tear occurred. In the days following the procedure, the patient claimed an improvement in night vision.

During the first visit at 3-month post-treatment, BCVA improved from 0.8 to 0.6 logMAR in the right eye and from 1.0 to 0.7 logMAR in the left eye, and nystagmus was significantly alleviated. Visual field mean deviation (MD) increased from 22.55 dB to −16 0.70 dB in the right eye and from −23.88 dB to −15.81 dB in the left eye with novel improvement (Figures 1A,B) although they were still in quiet low level. OCT showed no retinal edema (Figures 2A,B): FT was 155 μm in the right eye and 159 μm in the left eye (Figure 2B). Binocular FST results for white light improved from −1.905 to −5.013 log cd·s/m^2^ (0.95 to −30.13 dB); for blue light, from −2.361 to −5.770 log cd·s/m^2^ (−3.61 to −37.7 dB); and for red light, from −1.319 to −3.173 log cd·s/m^2^ (6.81 to −11.73 dB) (Table 1).

At 6-month post-treatment, BCVA, FT, and MD remained similar to the 3-month values. MD was −15.75 dB in the right eye and −15.07 dB in the left eye (Figure 1C); FT was 164 μm in the right eye and 152 μm in the left eye (Figure 2C). Fundus photography showed a round depigmented lesion usually indicated as chorioretinal atrophy (Sassen et al., 2026; Merle et al., 2025), measuring 0.5- papillary diameter (PD) around the injection site adjacent to the superior temporal vascular arcade in the right eye (Figure 2C). In the left eye, a round lesion of chorioretinal atrophy approximately 2 PD was visualized at the injection site alongside the superior temporal vascular arcade, with two patchy depigmented lesions each 1 PD in size located beside it (Figure 2C).

Considering the slow resensitization kinetics of treated rod photoreceptors that require prolonged dark adaptation to reach maximal sensitivity, an exploratory 8-h extended dark-adaptation FST test was performed exclusively at the 6-month visit, as referenced in a prior clinical report (Cideciyan et al., 2008). FST was −6.530 log cd·s/m^2^ (−45.3 dB) for white light, −7.116 log cd·s/m^2^ (−51.16 dB) for blue light, and −4.514 log cd·s/m^2^ (−25.14 dB) (Table 1), which may explain why the patient felt he could see better when he woke up in the morning.

The patient’s anterior segment remained essentially unchanged at 3- and 6-month post-treatment. Anterior segment examination showed no inflammation or other abnormalities: corneas were transparent, anterior chambers were of normal depth, iris texture was clear, pupils were round, and lenses were clear (Table 2).

At 22-month post-treatment, although the binocular FST was still maintained almost normal., the BCVA gradually declined to a level close to baseline (Table 2). No abnormalities were observed in the anterior segment. Fundus examination revealed coalescence and enlargement of the chorioretinal atrophic lesions (Figure 2D): Right eye: A nummular chorioretinal atrophy (arrow) of approximately 1 PD was visible around the superior temporal vascular arcade about 2 PD from the fovea, with scattered punctate atrophic lesions in the vicinity. Left eye: A horizontally oval depigmented lesion of about 6 PD was visible around the superior temporal vascular arcade at a location 1.5 PD from the fovea, with two patchy chorioretinal atrophic lesions of 1/3 PD and 2 PD, respectively located superior to it (Figure 2D). Note: The lesions in both eyes did not involve the macula/fovea till 22-month post-treatment (Figure 2D). The MD of the VF examination was −15.71 dB in the right eye and −12.09 in the left eye (Figure 1D); FT was 158 μm in the right eye and 160 μm in the left eye (Figure 2D) although the fixation losses rates, false positive and negative errors of VF examination also went up and reached to unreliable level at 22-month post-treatment (Figure 1D). We re-examined binocular FST. Encouragingly, under standard 2-h dark adaptation, binocular FST results for both eyes were similar to those at 6-month post-treatment and remained within almost normal levels. The FST values were −6.344 log cd·s/m^2^ (−43.44 dB) for white light, −6.976 log cd·s/m^2^ (−49.76 dB) for blue light, and −4.424 log cd·s/m^2^ (−24.24 dB) for red light (Table 1).

## 3 Discussion

Since the FDA approved VN in 2017 as the first and only gene therapy for biallelic *RPE65* mutations-mediated inherited retinal dystrophy, identifying patients with *RPE65* mutations has become a prerequisite for evaluating eligibility for *RPE65*-targeted gene therapy (Gao et al., 2021). VN is indicated for patients with sufficient viable retinal cells, particularly photoreceptors. Our previous preclinical work has shown that although delayed gene therapy can restore the function and partial morphology of remaining photoreceptors, early treatment leads to more effective overall retinal rescue and enhanced photoreceptor-mediated functional recovery (Pang et al., 2010; Nusinowitz et al., 2006; Pang et al., 2006; Li et al., 2011). Currently, most VN-treated patients are from Western populations. Due to small study sample sizes, there may be a risk of bias in clinical trials, resulting in populations with varying baseline characteristics (Birch et al., 2020). Although it is theoretically unaffected by race, sex, height, or weight, VN efficacy in Mainland Chinese patients has remained unknown.

This patient, confirmed to have LCA2 due to biallelic *RPE65* mutations, is the first Han Chinese patient in mainland China to receive VN gene therapy. OCT measurements showed sufficient viable rod and cone cells in both eyes, meeting VN eligibility criteria. Both eyes were sequentially treated by delivering VN into the subretinal space between the RPE and photoreceptor cells, around the superior macula. No obvious complications or adverse events occurred during the first couple of days following injections, or the first follow-up visit at 3-month after the procedure, consistent with previous reports of VN safety and efficacy (Sakai et al., 2024; Roman et al., 2022). The patient showed improvements in BCVA, VF, and binocular FST at 3-month after treatment. But depigmented lesions of chorioretinal atrophy developed at 6- and 22-month post-treatment visits although the macula/fovea was not involved and the FST and visual field are still maintained similar as previous report (Gange et al., 2022).

At 3-month post-treatment, binocular FST showed improvement compared to baseline. Blue light showed the greatest improvement, correlating significantly with the patient’s reported improvement in night vision.

Notably, at 22-month post-treatment, the patient’s binocular FST stayed nearly within normal ranges following a standard 2-h dark adaptation protocol, demonstrating sustained stable improvement in visual function. FST in decibels can best describe the physiological function of photoreceptors. Previous reports indicate that a white-light FST dimmer than −30 dB is rod-dominant; a red-blue difference of <3.1 dB suggests cone-dominant mediation; a difference of 3.1–19.3 dB suggests mixed rod-cone mediation; and a difference >19.3 dB suggests mainly rod-mediated thresholds (Birch et al., 2020; Sakai et al., 2024; Roman et al., 2022). In our patient, the pre-treatment binocular FST was 10.42 dB, indicating mixed rod-cone mediation. After treatment, the blue-red threshold difference was 25.97 dB at 3-month and 26.02 dB at 6-month post-treatment, both >19.3 dB, indicating that the binocular FST improvement was mainly rod-mediated. These results show that rod function improved more than cone function, although cone function also improved. The patient’s improvement was primarily in night vision, with only modest cone-function gains. Whether this relates to the injection site or the RPE65 enzyme has additional different recycle mechanism in cone requires further investigation.

BCVA usually serves as one of the primary endpoints for evaluating treatment efficacy in patients with visual dysfunction, but not in LCA2, which usually use FST and MLMT (Roman et al., 2022). Although the patient received treatment only in a small area above the macula, cone function indeed improved in some level. BCVA improved by −0.2 logMAR in the right eye and −0.3 logMAR in the left eye (increased about 1.5/2.0 times), and nystagmus was significantly reduced compared with baseline at 3-month post-treatment visit. Although these are subjective caregiver-reported observations, the patient claimed that he had never been able to recognize the facial details of his mother before; after treatment, he could clearly see her smile and facial details; moreover, he learned to read and became the rank #1 student in his class, achieving first place in one subject of his grade within 1-year post-treatment. These subjective observations still need validated by patient-reported outcome measures. We will pay more attention to mobility testing in the future follow up.

It is also worth noting that the patient claimed that the night vision remained normal, but the gradual improvement of the nystagmus from 3- to 6-month post-treatment stopped 1 year after treatment, which may indicate cessation of progressive central visual improving, and the daytime visual declined at 22-month post-treatment; the depigmentation lesions around the injection area also appeared at 6- and 22-month post-treatment follow ups (Figures 2C,D). Although we have the OCT data from the posterior pole, which did not show any chorioretinal atrophy around the macula/fovea, we did lack the OCT data of the possible chorioretinal atrophic lesions around the peripheral retina at the 6- and 22-month post-treatment follow-ups. But we do found the typical lesion of chorioretinal atrophy by infrared image (Figure 2D, middle column), which showed quantitative visualization of depigmented lesions of chorioretinal atrophy at 22-month post-treatment. From the previous Luxturna-related study, we know that chorioretinal atrophic lesions could be first detected around 1- to 22-month after Luxturna treatment (Fischer et al., 2026). About half of the treated eyes could appear chorioretinal atrophy; among them, many occurred within the subreinal blebs (Simoens et al., 2025); sometimes, chorioretinal atrophy could also be observed beyond the injection blebs; more than half of the treated eyes with chorioretinal atrophic lesions developed both within and beyond the subretinal blebs. The formation of such lesions may be multifactorial. Previous literature suggest that the atrophy could result from *RPE65*-replacement related dramatic changes from congenital hypo-metabolism or no RPE65 activity at all to hyper-metabolism in the treated RPE cells, which usually occurred in treated high activity-needed school-age patient eyes with good binocular FST restoration (Stingl et al., 2024; Stingl et al., 2023).In this patient, all lesions originated within the injection area, so local inflammatory response, AAV2 viral vector expression and injection-related factors cannot be excluded as contributing causes (Gange et al., 2022). Our previous studies showed that whole retina transfection with intravitreous injection of vector containing both AAV-*RPE65* and anti-apoptotic gene may lead to long-term rescue (Li et al., 2009; Li et al., 2008; Liu et al., 2023).

Summarizing previous results, we analyzed several possible factors for the further decline in cone function. First, the long-term efficacy from the subretinal injection-mediated gene therapy may be insufficient. AAV-based gene therapy may have limitations for long-term effectiveness; immune responses to the viral vector may affect intracellular expression, as confirmed in other gene therapy studies. If the interval between injections of the first and second eyes is too long, it may impact expression in the second eye and thereby affect recovery. Additionally, *RPE65*-related IRDs are mostly progressive diseases, so ongoing disease-related apoptosis in untreated visual cells beyond the injection area cannot be ruled out as a contributor to worsening. In summary, this case observation shows that Luxturna achieved similar visual improvements in this Han Chinese child with biallelic *RPE65*-related LCA2 as those from western patients. Following these findings, Luxturna was approved by Hainan Provincial Medical Products Administration (HPMPA) for clinical use at Hainan Boao He Eye Specialist Hospital under the special access pathway to import clinically urgent-need drugs in Hainan Boao Lecheng International Medical Tourism Pilot Zone, and offer insights for future research on gene therapy of IRDs in China.

## 4 Limitations and future perspectives

A major limitation of this study is that FST baseline was only measured under binocular viewing conditions without monocular testing from each eye by occluding the fellow eye because reliable monocular baseline data could not be obtained from this patient; binocular integrated threshold values are predominantly influenced by the better-functioning eye and cannot quantify independent visual function of each eye separately. The VF data at baseline and 22-month post-treatment were also not reliable perhaps due to the poor visual function of the patient. This study is limited to a single clinical case. Larger-sample and multicenter clinical observations are needed to further verify the efficacy and safety of voretigene neparvovec gene therapy in Chinese patients. In our study, only ocular manifestations, BCVA, and binocular FST were collected, while multi-luminance mobility test (MLMT) data were unavailable, and electroretinogram (ERG) testing was not performed because the patient declined the examination requests. No objective measurements of the child’s nystagmus were conducted, and relevant findings were only based on subjective observations by doctors and the child’s family.

We have previously shown that rods and cones respond to vector-encoded genes (Jacobson et al., 2012); however, dramatic improvements in binocular FST were not reflected in BCVA (Russell et al., 2017). The possible reasons are as follows: First, central visual function quantified by BCVA and peripheral rod function measured via FST rely on anatomically distinct visual transmission pathways. BCVA reflects high-acuity foveal vision mediated by densely packed cone photoreceptors, which transmit signals through the parvocellular pathway toward the central visual field in the primary visual cortex. In contrast, binocular FST mainly assesses extrafoveal and peripheral retinal rod function, which is relayed via the magnocellular pathway to cortical regions processing wide-field, low-light vision. Subretinal gene delivery was limited to the supramacular region rather than the central fovea, leading to partial restoration of peripheral rod signals but insufficient rescue of foveal cone function at the retinal level alone. Second, the poor cone function and related cone signal loss from birth—affecting both the eye and the visual cortex—may hinder the development of the visual cortex’s ability to integrate visual signals into coherent images (Levelt and Hübener, 2012; Cohen et al., 1999). Early visual development is a critical period during which the visual system is highly sensitive to environmental stimuli and capable of rapid, profound changes in response to visual experience (Levelt and Hübener, 2012; Cohen et al., 1999). Neural plasticity is heightened during this critical period, and the cortical network supporting vision is highly adaptive and shaped by sensory input or combined with electrical stimuli (Hubel and Wiesel, 1970; Polat et al., 2004; Polat and Sagi, 1994; Sharf et al., 2022; Park and Thompson, 2024; Raveendran et al., 2021). Evidence suggests that a continuous training based on RevitalVision (Polat et al., 2004; Polat and Sagi, 1994) can induce neuroplasticity, creating windows of opportunity for vision rehabilitation and improving visual function of the patients with glaucoma or amblyopia (Sharf et al., 2022; Park and Thompson, 2024; Raveendran et al., 2021). Therefore, different patterns of images to the visual pathway, particularly the occipital visual cortex, may be necessary. This will also be the focus of our follow-up research. If effective, this approach could benefit congenitally blind children after—or even before—gene therapy. For the present patient who received gene therapy, we have launched visual perceptual training intervention to further improve his visual function. Relevant research is currently ongoing, and we will report the follow-up outcomes in due course.

## Data Availability

The raw data supporting the conclusions of this article will be made available by the authors, without undue reservation.
